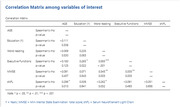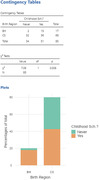# Is the countryside still inside? Exploring long‐standing effects of childhood rurality for dementia risk in the PROAME Study

**DOI:** 10.1002/alz.092316

**Published:** 2025-01-09

**Authors:** João Victor de Faria Rocha, Clarisse Vasconcelos Friedlaender, Kelle Luisa dos Santos Tomaz, Emma Patrice Ruppert, Aida Lourandes, Ana Paula Zacarias, Joanna de Castro Magalhães Assenção, Luciana Paula Rincon, Norton Gray Ferreira Ribeiro, Howard J. Rosen, Lea T. Grinberg, Francisca Izabel Pereira Maciel, Paulo Caramelli, Elisa de Paula França Resende, Cognitive and Behavioral Neurology Research Group (CogNeuro_UFMG)

**Affiliations:** ^1^ Universidade Federal de Minas Gerais, Belo Horizonte, Minas Gerais Brazil; ^2^ Faculdade de Medicina de Ciências Médicas de Minas Gerais, Belo Horizonte, Minas Gerais Brazil; ^3^ Escola Municipal Dr Julio Faria, Belo Horizonte Brazil; ^4^ University of California San Francisco, San Francisco, CA USA; ^5^ Memory & Aging Center, Department of Neurology, University of California in San Francisco, San Francisco, CA USA; ^6^ Faculty of Medicine ‐ Universidade Federal de Minas Gerais, Belo Horizonte Brazil

## Abstract

**Background:**

Dementia prevalence in Latin America is higher in rural than urban areas. This discrepancy may be explained by inequities over the lifespan in access to health and educational services. However, there is no evidence of potential adverse long‐standing effects of living in rural areas during childhood in current urban adults. This work investigates whether being born in rural areas (Rurality) increases exposure to dementia risk factors in adulthood among individuals who have emigrated to urban centers early in life.

**Method:**

130 individuals aged 40+ years were screened across 11 schools offering a late‐life educational program in Belo Horizonte. Participants underwent a comprehensive neuropsychological evaluation, sociodemographic interviews, literacy assessment, and serum neurofilament light chain (sNfL) exam. Participants were classified as originally from the metropolitan region (BH), or born in countryside regions (CS). The groups were compared in terms of sociodemographic features, cognitive performance, Dementia risk factors, and neurodegeneration.

**Result:**

108 met the inclusion criteria. Mean age was 58 (9.7) years, 71% women, and 90% non‐whites. CS (80% of the sample) and BH had similar sociodemographic characteristics. Cognitive performance was not significantly different. Concerning dementia risk factors, there was no difference in the frequency of sedentarism and depression (X², p = 0.38, for both), and for self‐reported hypertension, diabetes, dyslipidemia, smoking, alcohol consumption, hearing impairment, and severe head trauma. (X², p > 0.3 for all). Participants in the BH group were 4 times more likely to attend formal school during life, (p = 0.017), and had more years of schooling (p = 0.001). Education in childhood was positively associated with literacy level in adulthood (p = 0.024). Literacy level, but not education, was associated with global cognition, Executive functioning, and sNfL levels (p‐value = 0.003, 0.001, and 0.032).

**Conclusion:**

Decreased educational attainment in childhood was the main difference between the groups. This difference might be perpetuated over their lifespan in urban areas by a persistent disparity in basic literacy level. Our study suggests that low literacy/illiteracy might be an underlying mechanism linking rural origins to increased dementia risk, even among Brazilians currently in urban areas.